# Temperature-modulated separation of therapeutic cells, viral vectors, and exosomes using functional polymers

**DOI:** 10.1007/s44211-025-00785-x

**Published:** 2025-05-23

**Authors:** Kenichi Nagase, Hideko Kanazawa

**Affiliations:** 1 Graduate School of Biomedical and Health Sciences, Hiroshima University, 1-2-3 Kasumi, Minami-ku, Hiroshima City, Hiroshima 734-8553 Japan; 2 Faculty of Pharmacy, Keio University, 1-5-30 Shibakoen, Minato, Tokyo 105-8512 Japan

**Keywords:** Thermoresponsive polymer, Thermoresponsive interface, Temperature-responsive chromatography, Polymer brush, Regenerative medicine, Biomaterials

## Abstract

In recent decades, advanced therapeutic modalities such as therapeutic cells, viral vectors, and extracellular vesicles (exosomes), have emerged as effective therapies for intractable diseases. These therapeutic modalities produced through bioprocesses must be purified from contaminants. Effective separation methods are essential for optimizing therapeutic modalities. This review highlights innovative temperature-modulated separation methods enabled by the thermoresponsive polymer poly(*N*-isopropylacrylamide) (PNIPAAm). The design of PNIPAAm-modified interfaces plays a pivotal role in ensuring precise and efficient separation. We summarize the recent advancements in the application of temperature-modulated separation methods for cells, viral vectors, and exosomes, with a focus on the design of PNIPAAm interfaces.

**Graphical abstract:**

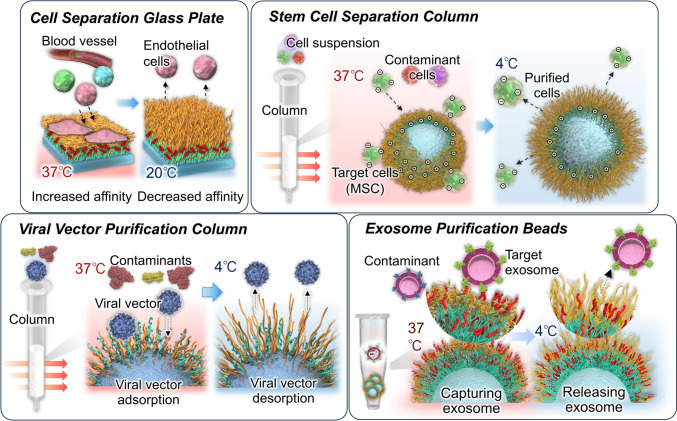

## Introduction

Biopharmaceuticals such as antibody drugs, cellular drugs, viral vectors, and exosomes have emerged as new therapeutic modalities in addition to conventional small-molecule drugs. Since these modalities are produced through bioprocesses, they must be purified to remove impurities. However, current purification processes for these modalities are expensive and may compromise drug activity. Therefore, there is a need for an efficient method to separate and purify these drugs at a lower cost while preserving their activity.

**Fig. 1:**
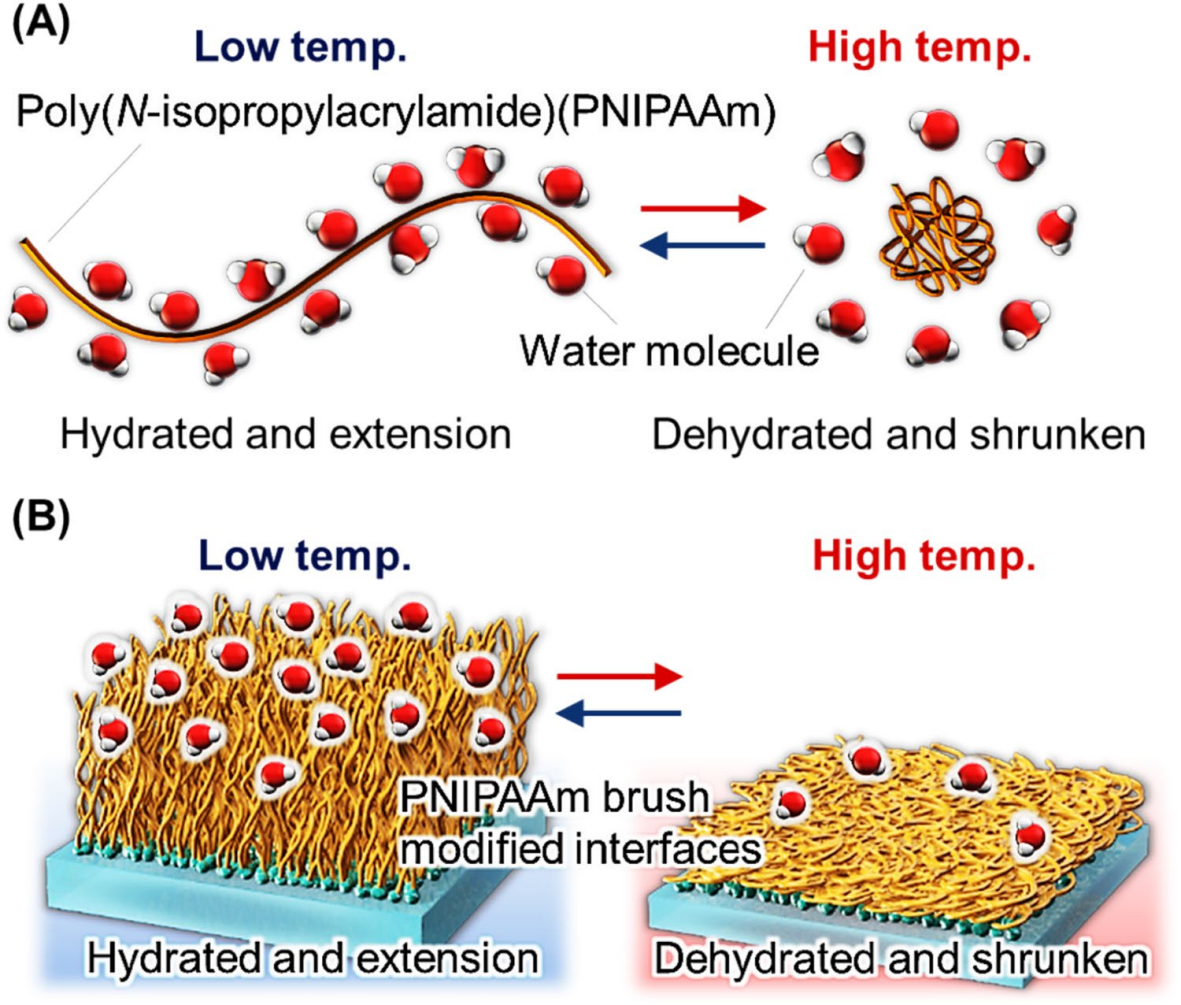
Illustration of **A** temperature-responsive poly(*N*-isopropylacrylamide) (PNIPAAm) and **B** PNIPAAm brush-modified interfaces

To address this issue, we developed a separation and analysis method using the thermoresponsive polymer poly(*N*-isopropylacrylamide) (PNIPAAm). PNIPAAm exhibits temperature-dependent transitions between hydrophilic and hydrophobic states owing to hydration and dehydration. This reversible behavior allows PNIPAAm to extend or shrink depending on temperature changes (Fig. 1) [1–7]. The thermoresponsive properties of PNIPAAm have been utilized in various biomedical applications. These include temperature-controlled drug and gene delivery systems [8–15], biosensors and bioimaging systems [16–23], nanoactuators [24–30], bioseparation tools [31–41], cell separation materials [42–48], and cell culture substrates for tissue engineering [49–67].

The thermoresponsive properties of PNIPAAm find application in temperature-responsive chromatography [68–73]. The chromatography system uses a PNIPAAm-modified stationary phase, where temperature changes regulate the interaction between the stationary phase and analytes. Hydration and dehydration of PNIPAAm-modified stationary phase with temperature results in shifts between hydrophilic and hydrophobic states. Hydrophobic analytes like steroids can be separated by temperature-responsive chromatography through hydrophobic interactions [68, 74]. Additionally, incorporating hydrophobic monomers into PNIPAAm enhances the hydrophobic interactions with the analyte. The enhanced hydrophobic interaction enables the separation of hydrophilic analytes such as insulin [75], amino acid phenylthiohydantoins [76], the benzoic acid family [77, 78] and benzodiazepines and barbiturates [79].

The introduction of ionic groups to PNIPAAm has enabled the development of temperature-responsive ion-exchange chromatography for separating ionic analytes. Specifically, temperature-responsive anion-exchange chromatography involves the integration of cationic monomers into PNIPAAm. This chromatographic technique can separate acidic biomolecules such as adenosine nucleotides and oligonucleotides through temperature-modulated electrostatic interaction [41, 80, 81]. Similarly, temperature-modulated cation-exchange chromatography incorporates anionic monomers into PNIPAAm. This enables the separation of basic biomolecules such as catecholamines and angiotensins [82, 83]. Temperature-responsive mixed-mode chromatography is another technique, which utilizes a combination of PNIPAAm-modified beads and ionic polymer-modified beads [84, 85]. This chromatography technique can be modulated via electrostatic interactions by changing the bead composition of the column. Temperature-responsive chromatography has been applied in therapeutic drug monitoring, particularly in hospitals, since it functions without organic solvents in the mobile phase [86–89].

Therefore, various temperature-responsive chromatography methods have been developed, emphasizing the need for designing modified thermoresponsive polymers for separating new medical modalities. This review summarizes the recent advances in separation technologies for therapeutic cells, viral vectors, and exosomes, along with the progress in the design of separation materials.

## Temperature-modulated cell separation with thermoresponsive ionic polymer brushes

Regenerative medicine is an effective approach for treating various intractable diseases [90–101]. These therapies are performed using cell suspensions or cellular tissue transplantation. Cells with therapeutic effects are typically mixed with contaminants, necessitating effective cell separation methods for regenerative applications. Various cell separation methods have been developed [102–115].

Centrifugation, which separates cells based on differences in specific gravity, is the simplest method and allows for large-scale separation of the cells. However, its low cell selectivity results in limited separation accuracy. Microfluidic devices offer an alternative approach, separating cells according to their size [109]. Here, fluid distribution is achieved by creating two types of microchannels, allowing the continuous separation of cells with different sizes.

More precise cell separation techniques include the use of antibodies labeled with fluorescent markers or conjugated to magnetic particles [102, 103]. Fluorescence-activated cell sorting (FACS) uses a fluorescence labelled antibody having affinity for target cells. Cells are fluorescently stained using these extracellular markers and then separated based on their fluorescence color or intensity. However, this separation method modifies the cell surface with fluorescent antibodies, resulting in the loss of cellular characteristics. Magnetic-activated cell sorting (MACS) has also been used as a precise cell separation method. MACS uses antibody-conjugated magnetic beads [103]. Magnetic beads are conjugated to the cell surface through interactions between the antibody and cell surface. The magnetic bead-modified cells are separated from the unmodified cells using magnetic force. However, the magnetic bead modification of cell surfaces also results in the loss of cell characteristics. Thus, FACS and MACS technologies involve cell modifications, which can alter their inherent properties and potentially diminish their therapeutic efficacy. Consequently, there is a growing demand for cell separation methods that preserve the integrity of the cell surface.

A promising alternative involves PNIPAAm-modified surfaces as a novel cell separation tool. The PNIPAAm-modified interface can control cell adhesion and detachment by temperature-dependent hydration and dehydration [49, 116]. Additionally, different cell types exhibit varying adhesion behaviors on PNIPAAm surfaces [117], making them efficient cell separation materials.

A PNIPAAm homopolymer brush-modified glass substrate has been used as a temperature-modulated cell-separation tool that does not require cell modification [42]. For example, vascular endothelial cells and myoblasts were separated based on their different cell desorption rates from the PNIPAAm brushes upon decreasing temperature.

Additionally, a thermoresponsive cationic random copolymer brush, poly(NIPAAm-co-*N,N*-dimethylaminopropylacrylamide (DMAPAAm)-*co*-*tert*-butylacrylamide)(tBAAm), has been used to separate mesenchymal stem cells (MSCs). MSCs have strong anionic properties, which can easily interact with the cationic polymer [44]. The selective adhesion of MSCs to the copolymer brush allowed for their targeted recovery, simply by lowering the temperature and inducing hydration and extension of the copolymer brush.

A thermoresponsive cationic copolymer brush has been prepared by random copolymerization of cationic and thermoresponsive monomers. This ensured a uniform incorporation of cationic groups into the thermoresponsive polymers. In temperature-responsive chromatography for protein separation and purification, packing materials prepared by block copolymerization (with bottom ionic segments and upper thermoresponsive segments) exhibited more effective protein adsorption and desorption than randomly copolymerized packing materials [34, 35, 118, 119]. This was due to the effective utilization of PNIPAAm shrinkage and extension. At high temperatures, the upper PNIPAAm segment shrinks, leading to effective protein adsorption onto the bottom ionic polymer brush. In contrast, at low temperatures, the upper PNIPAAm segment extends, preventing protein interactions with the ionic polymer. These results indicate that block-copolymer brushes are effective polymer structures for temperature-modulated separation of biomolecules.

**Fig. 2:**
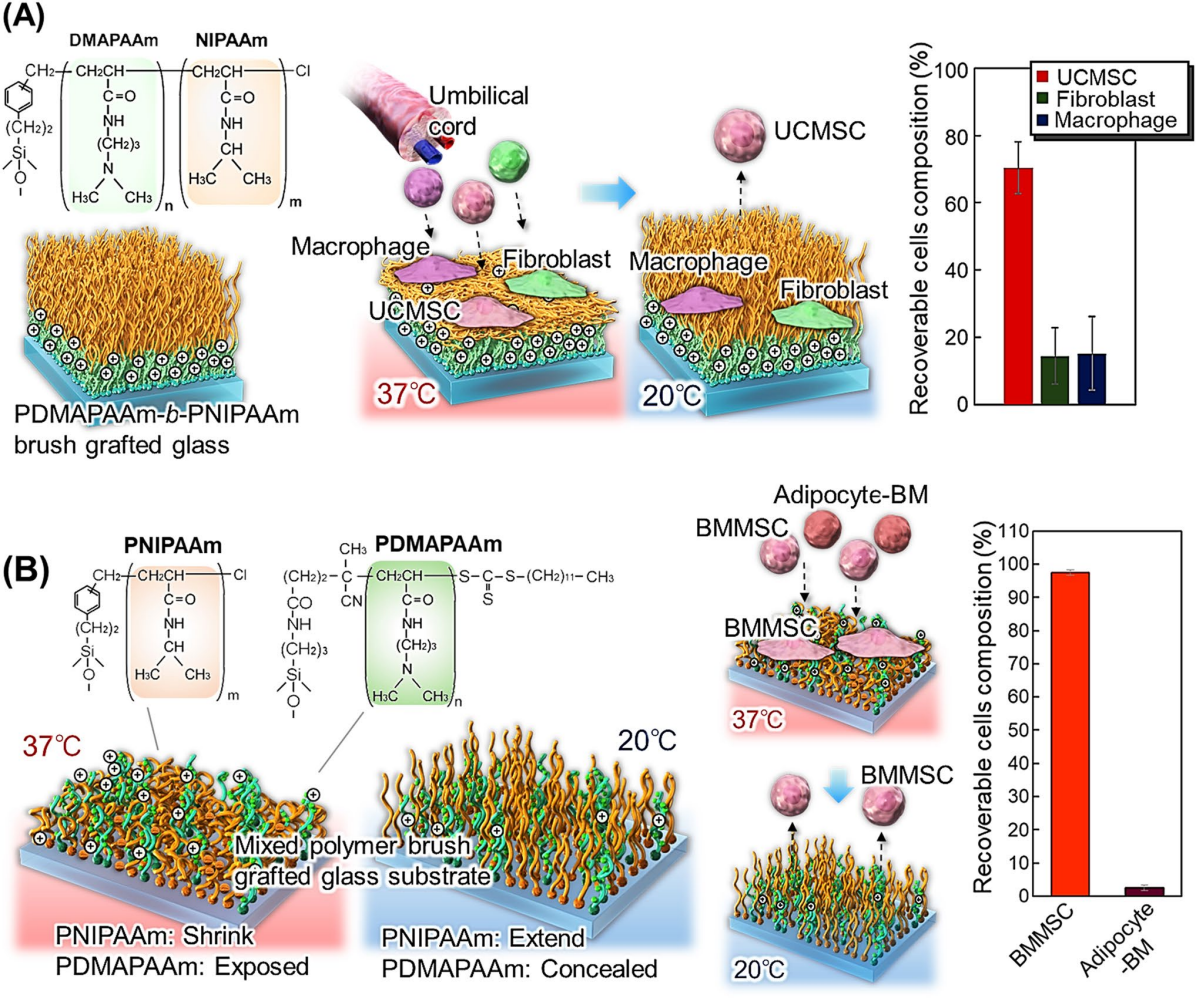
Temperature-modulated cell separation using thermoresponsive ionic polymer brushes. **A** UCMSC separation using a thermoresponsive cationic block copolymer brush-grafted glass substrate **B** BMMSC separation from differentiated adipocytes using a mixed polymer brush composed of PNIPAAm- and PDMAPAAm-modified glass substrates

Therefore, thermoresponsive cationic block copolymer brushes were investigated as separation materials for umbilical cord-derived mesenchymal stem cells (UCMSCs) (Fig. 2A) [120]. A poly(*N,N*-dimethylaminopropylacrylamide) (PDMAPAAm)-*b*-PNIPAAm brush was prepared on a glass substrate using two-step activator regeneration via electron-transfer atom-transfer radical polymerization (ARGET-ATRP) [121, 122]. These copolymer brushes have a cationic basal segment and a thermoresponsive upper segment. At 37 °C, the upper PNIPAAm segment shrinks, expressing the cationic property on the outermost copolymer brush. This enabled the adhesion of anionic UCMSCs to the copolymer brushes. Upon reducing the temperature to 20 °C, the adhered UCMSCs selectively detach due to the extension of upper PNIPAAm and weakened interaction between UCMSCs and the bottom cationic polymer segment. In contrast, fibroblasts and macrophages remain adhered to the copolymer brush even when the PNIPAAm is extended. Using the properties of the block copolymer brush, UCMSCs can be successfully separated from contaminant cells by temperature modulation [120].

Thermoresponsive anionic copolymer brushes have also been developed as vascular-cell separation materials [123]. This is because the anionic group effectively interacts with the vascular cells [124]. A poly(acrylic acid) (PAAc)-*b*-PNIPAAm brush was prepared on a glass substrate through a two-step ARGET-ATRP of the *tert*-butyl acrylate (tBA) and PNIPAAm, followed by deprotection of *tert*-butyl group of tBA [82, 83]. The preparation scheme was designed to prevent deactivation of the ATRP catalyst with anionic monomers during polymerization. The block copolymer brush has a basal anionic segment and an upper thermoresponsive segment. The block copolymer brush adheres to endothelial cells and smooth muscle cells at 37 °C owing to the dehydration and shrinking of the upper PNIPAAm segment. By reducing the temperature to 20 °C, the upper PNIPAAm segment gets hydrated and extended, leading to detachment of the adhered endothelial cells. On the contrary, smooth muscle cells remain attached to the copolymer brush even at 20 °C. This is due to the strong adhesive properties of smooth muscle cells from the possible interaction with the bottom anionic polymer segment. Using the properties of thermoresponsive anionic block copolymer brush, the endothelial and smooth muscle cells can be separated by simply changing the temperature.

Effective temperature-modulated cell separation has been achieved using thermoresponsive ionic block copolymer brushes. Mixed polymer brushes comprising two types of polymers have been developed for effective temperature-modulated protein separation [125]. Silica bead-packed column modified with mixed polymer brush PNIPAAm and PDMAPAAm exhibited temperature-modulated acidic protein adsorption. At high temperatures, PNIPAAm shrinks and PDMAPAAm is exposed, leading to effective adhesion of the acidic protein. While at low temperatures, PNIPAAm is extended and hydrated, leading to protein desorption.

Thermoresponsive cationic mixed-polymer brushes have been developed for the effective separation of MSCs (Fig. 2B) [126]. A mixed polymer brush composed of PNIPAAm and PDMAPAAm was developed on a glass substrate by the reversible addition-fragmentation chain transfer (RAFT) polymerization of DMAPAAm and subsequent ARGET-ATRP of NIPAAm. The mixed-polymer brushes exhibited temperature-modulated cationic properties. At 37 °C, PNIPAAm of mixed polymer brush shrinks and exposes PDMAPAAm, leading to increased cationic property. On the contrary, at 20 °C, extension of PNIPAAm conceals PDMAPAAm, leading to decreased cationic properties. Thus, at 37 °C, acidic bone marrow-derived mesenchymal stem cells (BMMSCs) selectively adhere to the mixed polymer brush because of enhanced electrostatic interaction. By reducing temperature to 20 °C, PNIPAAm hydrates and extends, thereby decreasing the interaction between BMMSCs and DMAPAAm. Thus, the adhered BMMSCs are detached from the mixed polymer brushes. In contrast, adipocyte differentiated from BMMSCs do not adhere to mixed-polymer brushes owing to their low adhesive activity. Based on this property, BMMSCs and differentiated adipocytes can be separated using a mixed polymer brush by varying the temperature.

## Temperature-modulated cell separation with thermoresponsive cell-affinity polymer brush

Peptide sequences with specific affinities for cells have been discovered and used in various medical applications [127–133]. For example, the Arg-Glu-Asp-Val (REDV) peptide is used to endothelialize the lumen of artificial blood vessels because of its specific interaction with vascular endothelial cells [132, 134]. Since these cell-affinity peptides interact with specific cells, they can be utilized as effective ligands for cell separation.

**Fig. 3:**
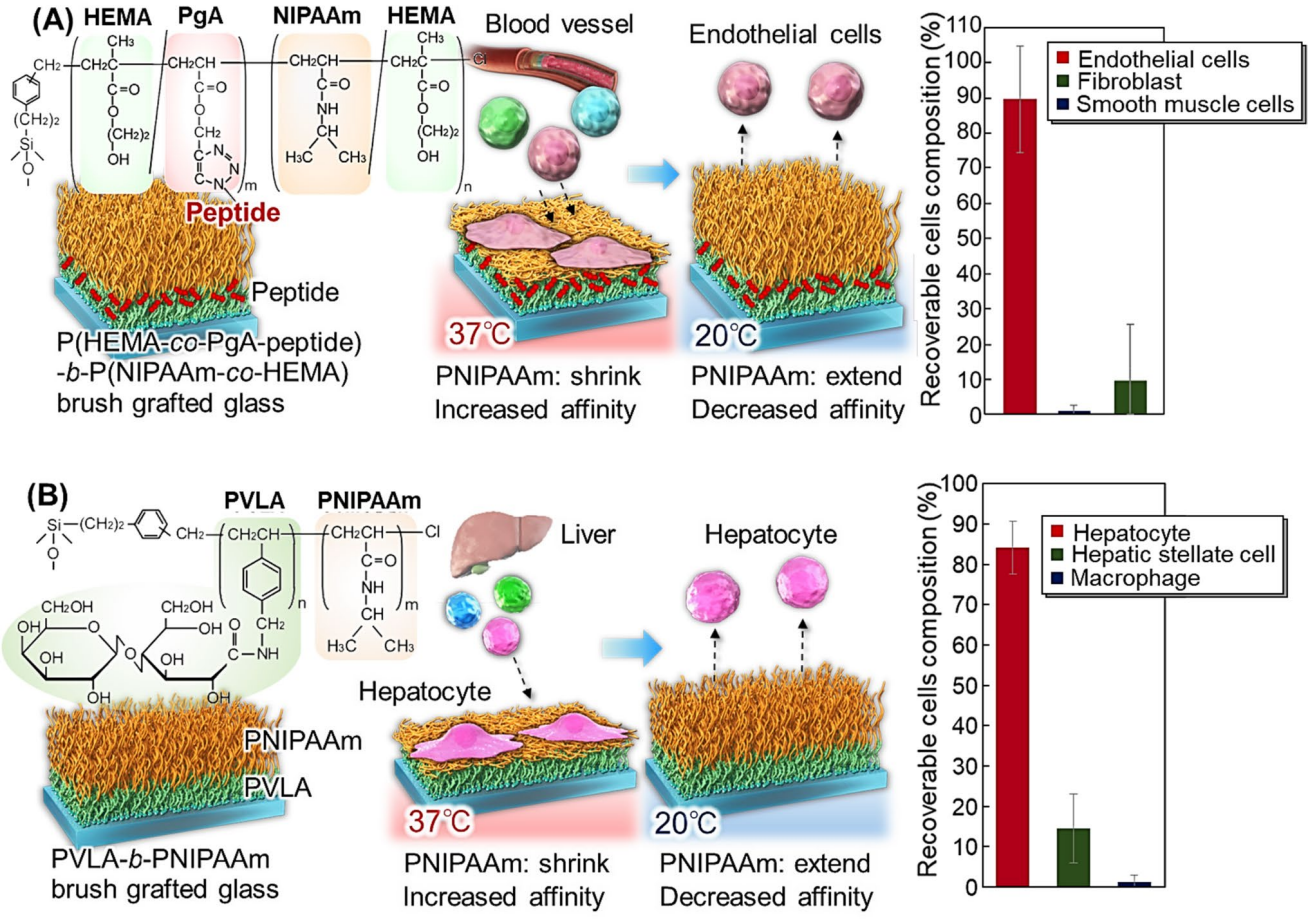
Temperature-modulated cell separation using thermoresponsive affinity polymer brushes. **A** Vascular cell separation using peptide-conjugated thermoresponsive block copolymer brush-grafted glass substrate and **B** hepatocyte separation using thermoresponsive glycopolymer block copolymer brush-grafted glass substrate

To improve the separation efficiency of endothelial cells, a cell-affinity peptide, REDV, is introduced into the thermoresponsive block copolymer brushes (Fig. 3) [135, 136]. A block copolymer brush composed of a bottom poly(2-hydroxyethyl methacrylate-*co*-propargyl acrylate)(P(HEMA-*co*-PgA)) segment and an upper P(NIPAAm-*co*-HEMA) segment was prepared using two-step ARGET-ATRP (Fig. 3A). Then, an Arg-Glu-Asp-Val (REDV) peptide with affinity for endothelial cells was conjugated to the bottom segment through a click reaction [137, 138]. The copolymer brushes exhibited a temperature-modulated affinity for target cells. At 37 °C, the upper segment shrinks, exposing the bottom segment conjugated with cell-affinity peptide at the outermost layer of the copolymer brush. This exposure leads to the effective adhesion of endothelial cells to the copolymer brush due to the affinity between the peptide and endothelial cells. By reducing the temperature to 20 °C, the upper segment gets extended and the affinity interaction between peptide and cells is reduced, leading to the detachment of endothelial cells. On the contrary, other contaminant cells, such as fibroblasts and smooth muscle cells, do not adhere to the copolymer brush at 37 °C. This occurs because the peptide in the copolymer has no affinity with fibroblasts and smooth muscle cells. Using this property, endothelial cells can be effectively separated from other vascular cells by simply changing their temperature.

A similar approach was used for poly(2-(2-methoxyethoxy)ethyl methacrylate) (PMEO_2_MA)-based copolymer brushes [136]. PMEO_2_MA-based copolymers have been investigated as functional materials for bioapplications requiring both thermo-responsiveness and bio-compatibility [139–146]. A P(HEMA-*co*-PgA)-*b*-poly(MEO_2_MA-*co*-HEMA-*co*-poly(ethylene glycol)methacrylate (PEGMA)) brush was prepared using two-step ARGET-ATRP. The REDV peptide was conjugated to the copolymer brush via a click reaction. The copolymer brush attaches to endothelial cells at 37 °C, attributed to the shrinkage of the PMEO_2_MA copolymer segment and exposed REDV peptide. In contrast, contaminant cells and fibroblasts do not adhere to the copolymer brush because the fibroblasts have no affinity for the peptide, and the PMEO_2_MA copolymer suppresses cell adhesion. By reducing the temperature to 20 °C, adhered endothelial cells are successfully recovered, leading to the separation of endothelial cells from contaminant cells.

Hepatocytes are important in regenerative medicine for the treatment of liver diseases[147–150]. Effective hepatocyte separation was achieved using the thermoresponsive glycopolymer brush (Fig. 3B) [151]. Glycopolymer poly(*N*-*p*-vinylbenzyl-*O*-*β*-D-galactopyranosyl-(1 → 4)-D-gluconamide) (PVLA) contains galactose moieties that interact with the asialoglycoprotein receptor (ASGPR) of hepatocytes [152–154]. Thus, PVLA can be used as a ligand for the effective capture of hepatocytes [151]. PVLA-*b*-PNIPAAm brush-grafted glass substrate using two-step ARGET-ATRP. At 37 °C, the upper PNIPAAm segment shrinks, and the bottom PVLA segment gets exposed. Thus, hepatocytes selectively adhere to the copolymer brushes through interactions between galactose and the ASGPR of hepatocytes. In contrast, hepatic stellate cells do not adhere to the copolymer brushes owing to their negligible affinity for the bottom glycopolymer. Other contaminant cells, such as macrophages, can adhere to the copolymer brush because of their strong adhesive properties. By reducing the temperature to 20 °C, the upper PNIPAAm segment extends and the interaction between galactose and ASGPR of hepatocytes is reduced. This leads to the successful recovery of hepatocytes from the copolymer brush. In contrast, adhered macrophages do not detach even at low temperatures, which is attributed to the strong adhesion properties of macrophages. Using the thermoresponsive properties of block-copolymer brushes, hepatocytes were separated from contaminant cells, macrophages, and stellate cells. Additionally, induced pluripotent stem (iPS) cell-derived hepatocytes are purified from undifferentiated iPS cells using a PVLA-*b*-PNIPAAm brush with a simple temperature change. iPS cell-derived hepatocytes adhered to the PVLA-*b*-PNIPAAm brush because they have an ASGPR. In contrast, iPS cells do not adhere to PVLA-*b*-PNIPAAm brush. By reducing the temperature to 20 °C, the upper PNIPAAm segment extends and the interaction between galactose and ASGPR of iPS cell-derived hepatocytes is reduced. This leads to the successful recovery of iPS cell-derived hepatocytes from the copolymer brush. Using the thermoresponsive properties of block-copolymer brushes, iPS cell-derived hepatocytes were separated from iPS cells.

## Temperature-modulated cell separation column for effective stem cell separation

The developed PNIPAAm copolymer brush-modified glass could separate cells simply by changing the temperature. However, cell separation is limited by the small surface area of copolymer-modified glass substrates. To improve cell separation performance, cell separation columns using PNIPAAm copolymers have been investigated [155–158].

**Fig. 4:**
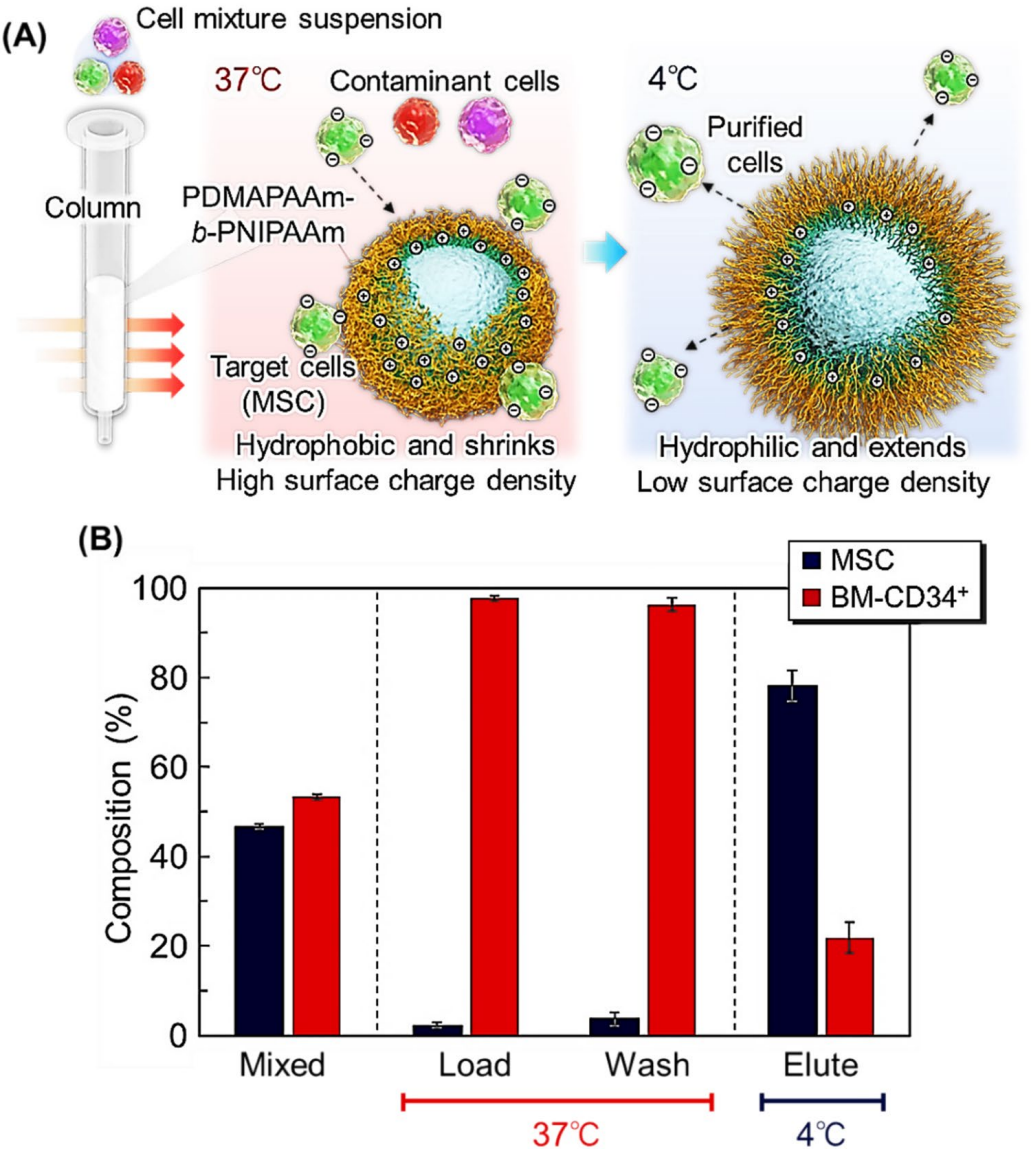
Temperature-modulated cell separation column. **A** Illustration of the cell separation column using thermoresponsive cationic block copolymer brush-grafted beads and **B** cell composition at each fraction

A temperature-responsive cell separation column was developed for bone marrow-derived MSC using PDMAPAAm-*b*-PNIPAAm brush-modified beads as the packing material (Fig. 4) [157]. Silica beads with a diameter of 150–210 μm were used as base materials because a relatively large bead diameter was required for cells to pass through the bead-packed layer. PDMAPAAm-*b*-PNIPAAm was modified via two ATRP steps. Because MSCs exhibit strong acidic properties, the cationic polymer PDMAPAAm functions as an effective ligand for capturing MSCs [157]. The copolymer-brush-modified beads are packed in syringe-type columns. At 37 °C, MSCs are introduced into the column. The upper PNIPAAm segment shrinks and gets dehydrated, exposing the bottom PDMAPAAm layer as the outermost layer of the beads. MSCs are negatively charged compared to other contaminant cells [157] and are adsorbed onto copolymer brushes through electrostatic and hydrophobic interactions. In contrast, the contaminant cells, BM-CD34^+^, are not adsorbed onto the beads owing to weak electrostatic interaction compared to the interaction between MSCs and PDMAPAAm. Thus, BM-CD34^+^ will pass through the column even at 37 °C. By reducing the temperature to 20 °C, the PNIPAAm segment gets extended and hydrated, reducing the electrostatic and hydrophobic interactions and causing the adsorbed MSC to desorb from the copolymer brush. Using this column, MSCs can be successfully separated from the contaminant BM-CD34^+^ cells by simply changing the column temperature. In addition, recovered MSCs maintained their proliferative and multilineage differentiation abilities.

A similar cell separation column was developed for the effective separation of adipose tissue-derived MSCs [158]. Unlike the separation columns for bone marrow-derived MSCs, silica beads with large diameters (212–250 μm) are used as base materials because the adipose tissue-derived MSCs have larger diameters compared to that of bone marrow-derived MSCs [158]. The PDMAPAAm-*b*-PNIPAAm brush was modified on silica beads through two ATRP steps, and the prepared beads were packed into a syringe-type column. The column retained adipose tissue-derived MSCs at 37 °C due to their adsorption on the PDMAPAAm-*b*-PNIPAAm brush through electrostatic and hydrophobic interactions. In contrast, contaminant Jurkat cells pass through the column because the electrostatic interactions between Jurkat cells and the copolymer are relatively weak. By changing the column temperature to 4 °C, MSCs are eluted from the column. These get desorbed from the copolymer brush due to reduced electrostatic and hydrophobic interactions. Using this column, adipose tissue-derived MSCs are separated from the contaminant cells by simply changing their temperature. Furthermore, the recovered MSC maintained their viability, proliferation, and differentiation abilities compared to those before cell separation using the column. These results indicated that the cell separation column.

## Temperature-modulated separation of viral vectors

**Fig. 5:**
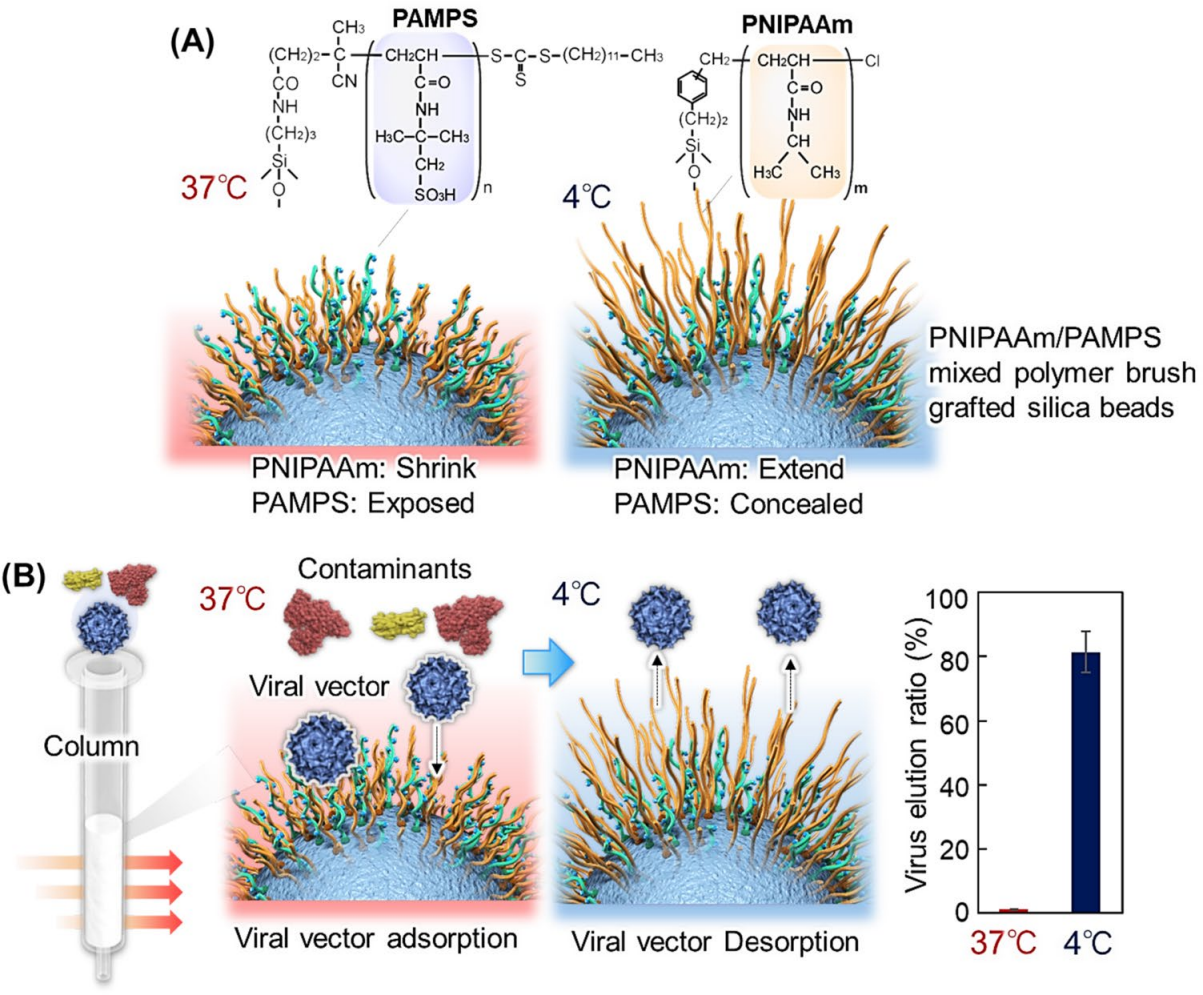
Temperature-modulated viral vector purification. **A** Illustration of packing materials for the purification column and **B** purification procedure and virus elution at two temperatures

Viral vectors are used for gene therapy because of their ability to deliver nucleic acids to target cells via transfection [159–161]. During the production of viral vectors, viral vector-producing cells are cultured, and the cells produce viral vectors in cell culture medium. Contaminants in the culture medium must be removed from viral vectors. Various purification methods for viral vectors have been developed [162–167]. The purification of viral vectors using cesium chloride density gradient centrifugation and column chromatography has been extensively investigated as effective separation methods [166, 167]. However, to improve the production efficiency of viral vectors further, effective viral vector purification methods using simple procedures are urgently required. Thus, viral vector purification methods using PNIPAAm have been investigated [168]. Adeno-associated virus type 2 (AAV2) vectors are particularly useful because of their stability, versatility, and non-pathogenicity [169–171]. Therefore, an AAV2 vector purification column was developed using PNIPAAm-based mixed-polymer brush-modified beads (Fig. 5) [168]. A mixed polymer brush composed of poly(2-acrylamido-2-methylpropanesulfonic acid) (PAMPS) and PNIPAAm served as an effective ligand for capturing viral vectors. During AAV2 infection, the AAV2 vector recognizes heparan sulfate proteoglycans on the cell surface [172–174]. Thus, PAMPS anionic polymers with sulfonic acid groups can interact with AAV2. Silica beads (40–64 μm) are used as base materials. Two types of initiators, a radical polymerization initiator and an ATRP initiator, are immobilized on the silica beads. PAMPS is grafted onto the silica bead surface via RAFT polymerization, and PNIPAAm is grafted onto the beads via ATRP, resulting in the formation of a mixed polymer brush. The beads are packed into a syringe column. AAV2 is introduced into the column at 40 °C, and AAV2 gets adsorbed onto the mixed polymer brush. At 40 °C, PNIPAAm shrinks, and PAMPS is exposed to the outermost layer of the mixed polymer brush, leading to effective adsorption of AAV2 on the mixed polymer brush. By reducing the temperature to 5 °C, adsorbed AAV2 is desorbed. At 5 °C, PNIPAAm is extended, and PAMPS gets concealed. Thus, the interaction between PAMPS and the viral vector is reduced, leading to the desorption of AAV2 from the mixed polymer brush and the elution of AAV2 from the column. Based on the properties of the mixed polymer brushes, AAV2 is successfully separated from bovine serum by varying the temperature. Furthermore, the recovered AAV2 strain retains its infectivity.

## Temperature-modulated separation of exosomes

Recently, exosomes have attracted considerable attention as potential diagnostic markers and therapeutic agents for cancer. Exosomes are small vesicles (40–100 nm) composed of endosomal cell membranes attributed to derived cells. Exosomes contain nucleic acids such as messenger RNA and microRNAs, and various proteins are attributed to their derived cells. Thus, exosomes can be used as diagnostic markers because they contain nucleic acids that are derived from cells [175, 176]. In addition, the administration of exosomes has been shown to have therapeutic effects in a variety of diseases, owing to the presence of nucleic acids and proteins [177–182]. Exosomes are secreted from cells and must be purified from cell culture media, which often contains various foreign substances. Various exosome purification methods have been developed, including ultracentrifugation, size exclusion, and affinity separation [183–187]. However, these separation methods are limited by complicated procedures, low selectivity, and reduced activity. Therefore, effective exosome purification methods that involve simple procedures and preserve exosome activity are urgently required.

Recently, innovative exosome isolation methods have been developed [188, 189]. The T cell immunoglobulin domain and mucin domain-containing protein 4 (Tim4), which specifically bind phosphatidylserine present on the surface of exosomes, were used for exosome purification [188]. In this method, magnetic beads conjugated with Tim4-Fc protein are added to exosome-secreted culture medium. Exosomes adhered to the beads via a Tim4-Fc-phosphatidylserine binding process in the presence of Ca^2+^ ions. Later, the bound exosomes are released from the beads using an elution buffer containing the chelating agent, ethylenediaminetetraacetic acid.

Further, exosome isolation methods using net-charge invertible curvature-sensing peptides (NIC) have been developed [189]. Curvature-sensing peptides recognize exosome vesicles by binding to lipid-packing defects on the highly curved exosome membranes. Additionally, the NIC design ensures efficient capture and release of the exosomes with pH changes. The NIC is modified on a resin and added to an exosome suspension maintained at pH 6.0 to bind the exosomes to the resin. Later, upon using a buffer having pH 10.0, the bound exosomes can be released.

**Fig. 6:**
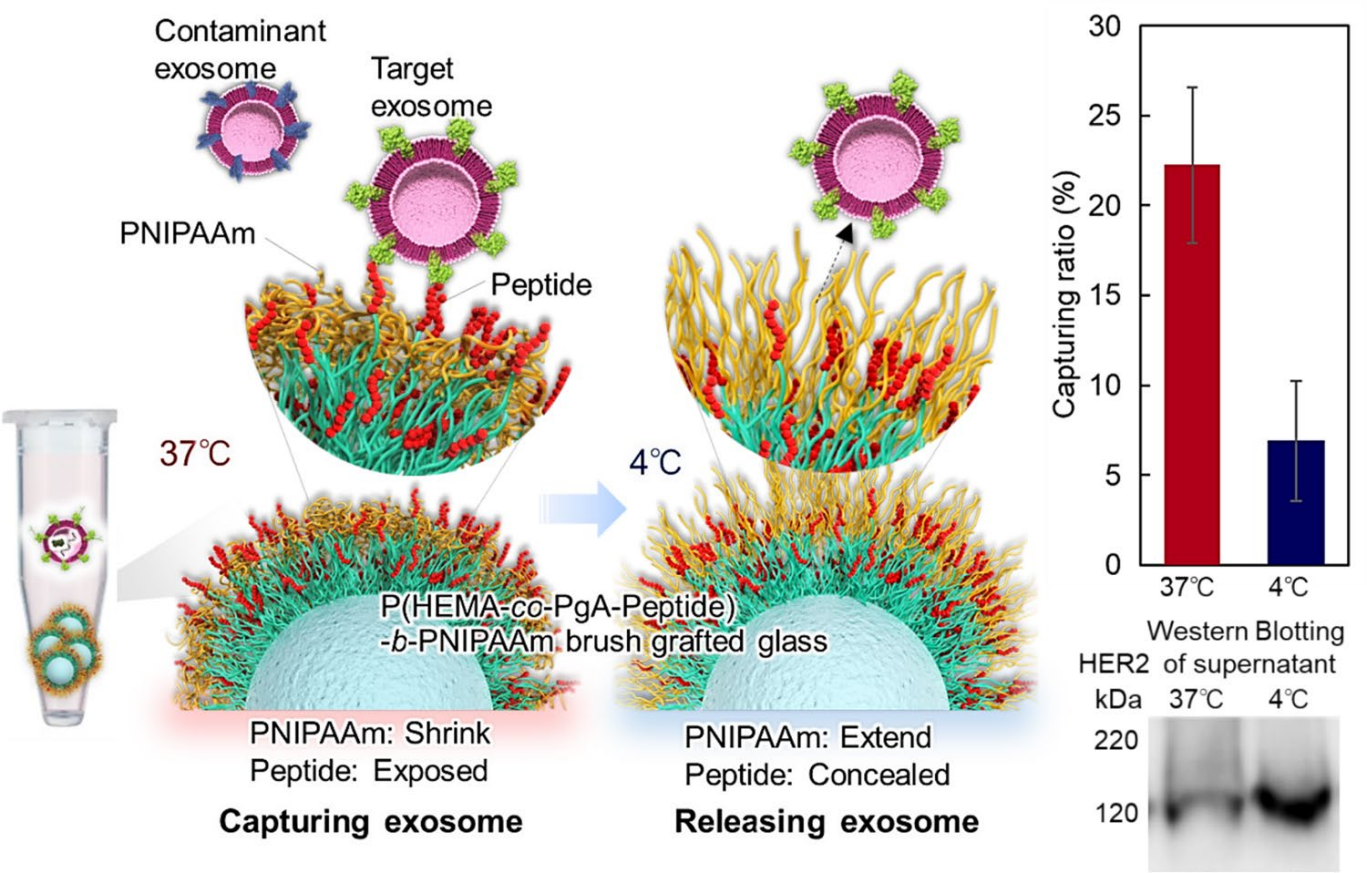
Temperature-modulated capture of exosomes using a thermoresponsive block copolymer brush with affinity peptides

We developed an innovative exosome purification method using PNIPAAm by simply changing the temperature (Fig. 6) [190]. Nonporous silica beads (diameter 4.0 μm) were used as base materials. First, a P(HEMA-*co*-PgA) brush is grafted onto the silica beads as a peptide conjugation segment during the first ATRP. To these grafted beads, PNIPAAm brush is grafted via a second ATRP step. Peptides having exosome affinity are then conjugated to the propargyl group in the bottom segment through a click chemistry reaction. After incubating the prepared beads and the exosomes derived from SK-BR-3 cells in a microtube at 37 °C, the exosomes are captured on the copolymer brush-modified beads. At 37 °C, the PNIPAAm segment shrinks and exposes the peptide-conjugated bottom segment on the outermost surface of the block copolymer brush. This leads to the effective capture of exosomes on the copolymer brush. Upon decreasing the temperature to 4 °C, the captured exosomes are released from the block copolymer brush. This is attributed to the extension of PNIPAAm, which reduced the peptide-exosome affinity. Hence, target exosomes can be purified by using these beads at different temperatures.

## Conclusions

This review summarizes innovative separation methods for new therapeutic modalities such as cells, viral vectors, and exosomes. It also highlights the design of thermoresponsive polymers. MSCs and endothelial cells can be effectively separated using thermoresponsive ionic block copolymer brush-modified glass substrates. These substrates regulate ionic properties with temperature changes. A mixed polymer brush composed of PDMAPAAm and PNIPAAm enables MSC separation from adipocytes using temperature modulation. In temperature-modulated cell separation, cell-affinity peptides and glycopolymers are introduced into block copolymer brushes. This enhances the affinity between copolymers and target cells. For effective MSC separation, cell separation columns have been developed using silica beads modified with thermoresponsive cationic block copolymer brush. A temperature-modulated viral vector purification column has also been developed using a mixed polymer brush composed of PAMPS and PNIPAAm. Additionally, functional microbeads incorporating block copolymer brushes and affinity peptides allow for temperature-modulated exosome capture and release. These innovative bioseparation methods hold significant potential for biopharmaceutical production and separation.

## Data Availability

Data are available from the corresponding author upon reasonable request.
